# Action and Uses of Alcohol

**Published:** 1863-07

**Authors:** J. R. Lothrop


BUFFALO

Medical and Surgical Journal

VOL. II. JULY, 1863. NO. 12.

### ORIGINAL COMMUNICATIONS.

ART. I.—*Action and Uses of Alcohol.*—By Dr. J. R. Lothrop.—*Read before the Erie County Medical Society.*

[Published by vote of the Society.]

As a subject fraught with great interest and importance in a moral and physical sense, I propose to call attention to some considerations upon the action and uses of alcohol. This is of peculiar interest at this time from the great efforts made to prove its use in all quantities always and invariably destructive and never constructive, and because there is a fear on the part of many good men, both professional and non-professional, that in therapeutics there is at the present time a tendency to excessive alcoholism. This tendency of therapeutics to oscillate from one extreme to another, is apparent to any one who studies in the past the radically opposite directions of the medical mind of different periods. To go no further back than the period to which the memory of many now living extends, we find methods of treatment at variance with those which now all judicious medical men adopt and pursue. Reduction where now we find stimulation. By the one, disease was deprived of its pabulum that it might be under the necessity of evacuating the body, by the other, support is given to the material fabric against the wasting power of disease. Now each period would speak in no gentle terms of the practice of the other—one would stigmatize as incendiary what the other considers sustaining. Each would consider salutary, nay assential, a practice which the other would condemn as destructive. Indeed they who formerly with fear and trembling, lest they were furnishing phlogistic material to kindle anew the smothered flame of disease, ventured upon stimulants at the decline of an acute disorder, might well be astounded at the giving of a quart or two of alcoholic liquor daily, at the very outset. The practice and theories of Dr. Todd in pneumonia and other acute diseases, we can easily imagine would startle one bred in the olden school.

It is worth while then to enter into some examination of the grounds upon which our practice rests, and ascertain if we can whether we are not tending to an extreme stimulation. At least let us make the best statement we can of the advantages of our method. If it has a rational foundation it will bear scrutiny. This involves far the better understanding of the subject—first, the consideration of the action of alcohol upon the body in health; afterwards something may be said of its uses.

In the outset I wish to avoid the moral issues connected with the subject. The benefits of teetotalism, or the deplorable mischiefs of excess as affecting the social or the spiritual good of the human race, is not properly a question to be entered into here. We know with how much hesitation as a profession we should shrink from the heavy responsibility of seeming, in the discussion of a scientific question, to advocate the use of a stimulant by allowing it to be productive of any benefit, which has been at the bottom of such deep woe to the human family. Advantages it has as well as dangers, and while reformers may and we hope will by painting the latter in broad lights, frighten those liable to excess out of its use, we are unwilling that those who need alcohol, and not reform, should by fear of the dangers be made sceptical of its advantages. If alcohol gives to man a rational pleasure without injury to his health, it might justify something urged in its favor. But if its moderate use makes man better fitted for the duties of life, if it makes him stronger, healthier, and therefore more cheerful and more useful, words of commendation are not only justified, but fitting to be spoken. For the perfection of the body has much to do with the perfection of the mind, and I think we may have much sympathy with the enjoyments of the senses in all innocent ways, if men are thereby made happier and in consequence better. Many of the moral maxims of the world are tinged with infirmities which an unsound body has imparted to the operations of the mind.

But this brings the question to the issue which we wish to consider, viz: what is the action of alcohol when taken into the body? Does it act as a poison always, and in all quantities, as some maintain, and therefore injuriously? Is it in any sense food, as others declare, and therefore beneficial? Or, is it merely a stimulant, giving a temporary exaltation, to be followed by exhaustion; in other words acting merely as a spur upon the horse, giving temporary increased activity at the expense of power? These questions it is needless to say, it is extremely difficult to determine, as there is not an agreement among physiologists upon them. Dr. Carpenter regards it as a poison in all quantities, while Dr. Todd regards it as food to the nervous system. Dr. Carpenter would place it on the list of medicines with chloroform or opium or ether, only to be resorted to in unusual conditions, while Dr. Chambers classes it as accessory food, with salt, sugar, tea and coffee, articles of daily domestic consumption. If we examine the question as presented by experimental physiology we shall put ourselves in possession of all the facts which our present knowledge affords.

In examining the question of its action we must not lose sight of what may be called its double action in different quantities. Like many other valuable agents its action differs not only in degree, but in kind, according to the different amounts taken. We cannot assume that what is true of a large quantity is true of a smaller, in a minor degree. A large dose we know to be poisonous, it would be fallacy to argue that a small one was poisonous also in a proportionate degree. If we were to reason in this way, or to lose sight of the fact that quantitative difference often make a qualitative difference, we should be often led into error. This difference in kind and not merely in degree, in different quantities, is very familiar to us not only among medicines, but the things necessary to life, as light and heat, nay even food itself. If we were to adopt any argument from their effects in excess, as is often done in the case of alcohol, it would be worthless. Among medicines arsenic is a poison in excess and destroys life; but in small quantities it imparts vigor to the body, freshens the complexion and increases its beauty. Strychnine kills almost with the rapidity of lightning, but in small quantities it is a valuable tonic. So it is with prussic acid, and measurably so with opium. We might adduce other instances. Heat, essential to organic health, in excess is actively destructive. Oxygen in excess is as actively poisonous as prussic acid. Light which is grateful to the eye, and a necessary stimulus, causes blindness in excess. How often in nature do we find a capacity for good associated in the same agent with a capacity for evil, and the one somewhat a measure of the other.

Quantity makes the difference and determines to one or the other. With the benefits of heat, light and electricity, what destructive capacities are associated. If the effect of alcohol is injurious in a large and beneficial in a small amount, it resembles a large class of agents which do good in moderation and harm in excess. There is no denial of the great injury arising from excessive use, but the same injury may arise from over-doses of many other things in themselves essentially beneficial. The line of argument which maintains that alcohol is essentially poisonous in all quantities, because it is in large, would be equally valid against tea and coffee, and even in some degree against beef and mutton.

The first question which demands examination in connection with the action of alcohol relates to its absorption and its escape from the body. When taken into the stomach in a concentrated from it is not as rapidly taken into the blood as when diluted with water. In fact it produces pathological changes such as congestion and hyperæmia, which interfere with its rapid absorption. This is an effect partly of its abstraction of water from the membrane of the stomach, partly of its power to harden tissues by producing coagulation of albumen, and partly of its action as an irritant. When mixed with water it undergoes rapid absorption by the stomach, passes into the blood, localizes itself by a sort of elective action in certain organs and tissues, and is then eliminated in various ways, but chiefly by the kidneys and the lungs, more by the former than the latter. In its course through the body it probably penetrates all tissues, but it accumulates by a sort of affinity in certain parts of the body. These parts are the brain, liver, and blood, but little accumulating in the muscles. The nervous tissue of the brain receives the largest proportion, next in order comes the liver, next the blood. We might infer this from the well known pathological changes which take place in these organs and in the blood. We know how frequent are mania, epilepsy and paralysis, cirrhosis, and defibrinated blood in those who use alcohol in excess. The escape from the body begins very soon after alcohol is taken, and is in the main very rapid; but though the larger proportion is eliminated in a short time, yet some portion remains for a considerable time, and is apparent to processes for its detection. It probably remains in the system as long as any of its effects are apparent. The most recent experiments show that while the effects continue they are due to its presence in the body, but that they are less and less as excretion goes on, and finally disappear with the elimination of the cause from the system. They do not continue after it has left the body, and are in direct proportion to the quantity taken.

The most striking point connected with its elimination is, that it leaves the body without having undergone chemical decomposition. Alcohol taken into the body escapes from the body as alcohol, not as carbonic acid and water, as is maintained by those who claim that it is respiatory food. And not only does it escape unchanged, but it is probable that the whole amount taken escapes, no portion being left in combination with the tissues. By careful experiments most of it can be recovered in its original form. Closely hunted in its progress through the body, and at the avenues of its escape, it yields itself up in almost undiminished quantity, In what manner then can it act as food for the tissues, or in any sense as respiratory food, offering its hydrogen to oxygen to form water, and its carbon to form carbonic acid, and thus saving the tissues from active destruction by combustion in respiration?

Whatever actions or changes it effects, they are brought about while it remains itself unchanged. This fact however does not in any manner go to disprove either the action or the beneficial agency of alcohol upon the human body, it only conflicts with theories of the manner of its action. Other substances, whose power for good is not a matter of conjecture, leave the body unchanged. Many medicinal substances are eliminated unchanged. Iodide of potash and chlorate of potash appear without change in the urine, so do mercury, and arsenic, and lead. Iron exists in the blood as iron. That all these substances exert a beneficial influence is a matter of daily experience, but it is by no means easy to state in what manner they affect it. Nay, water itself, the great promoter of metamorphosis of tissue, leaves the body as water; yet it is essential to life. If we argue that the actions which have been attributed to alcohol, predicated upon the fact of a chemical change in the body, cannot take place because it does not undergo decomposition, the reasoning is entitled to some weight. But if we assume that its action cannot be of any very great importance in the various processes of tissue formation and change which are constantly going on in the body, because it passes through it as alcohol, we should arrive at a conclusion which would overthrow what we consider well ascertained facts in regard to many efficient and useful medicines, water, and in some degree food itself.

These arguments are merely designed to meet the statement that alcohol can have no great benefits because it is not changed in the body. We can give to them their proper value, and readily see whither they lead. But whatever its action, we cannot escape the conclusion that alcohol belongs to the class of non-assimilative bodies, not directly nutrient to the body and working its changes by presence, or as a local excitant, and not by combination.

But we may somewhat farther consider the question, is alcohol food, that is, in what sense is it food? Does it directly act as a nutrient to the tissues, or indirectly by arresting destructive changes in the body? Dr. Todd maintains that it is "the appropriate pabulum" of the nervous tissue, in other words "it is a form of aliment appropriate to the direct nourishment of the nervous system, and to its preservation; its special adaptation to this system gives it an immediate exciting power superior to any other kind of food." But still though more exciting, i. e. more rapid, it acts like other forms of food. It is as much a special aliment to the nervous tissue as albumen is to the muscular. This theory cannot be true if alcohol is a non-assimilative body, and I think, in consideration of what has been already said, we may doubt his statement, that if taken only in quantity proportionate to the wants of the system, it will not be apparent by any process of elimination, i. e. it will not even be perceptible in the breath. Alcohol invigorates the nervous system—so do tea and coffee, so do moderate study and exercise in the open air, a ride on horseback, or a mountain walk, and like it, in excess, they weary and exhaust. But there is no reason to suppose that directly they furnish one particle of nutriment to the brain. We do not doubt their benefits, but they cannot be wrought in the manner assumed by Dr. Todd. But if we admit that it is food, and that in small quantity it repairs the brain like other food, it will not hold true of large quantities, for then its action is like other causes which waste rather than nourish the nervous system, viz: over-study, mental anxiety, excessive use of tobacco. Of the two, Dr. Carpenter's theory seems more reasonable, that alcohol is a suicidal instrument to the nervous system, stimulating it to its own destruction; if we are left to the necessity of adopting either.

Alcohol in its course through the body does not act upon it like other substances which we know to be directly alimentary. These are decomposed so that they do not appear again in the same form. Starch and sugar do not appear in the excretions as starch or sugar, at least in health, nor beef, or more properly albumen, as beef or albumen. Their ultimate elements appear in the excretions as carbon in that of the liver and lungs, nitrogen in that of the kidneys, after having performed an important part in the repair of the body. These substances undergo changes of decomposition which fit them for this important office, and we can readily understand how by these chances they replace the elements lost by the metamorphosis of tissue, and substitute new for worn out and effete material.

The fats, important substances of the class of hydro-carbons, among which alcohol is ordinarily placed, do not differ from alcohol by merely acting more slowly. They are, it is true, mostly absorbed unchanged in the body, being stored away in the interstices of the organs to favor their easy movement, and to give roundness and beauty to the human form.—They abound in the nervous tissue, and are therefore concerned in the intellectual phenomena, i. e. as matter, they perform some office in the operations of the mind. But though existing unchanged as alcohol does, they serve evidently some more ultimate purpose, since they are not excreted in the same form. They have some agency in nourishing the nervous system, and do actually repair nervous waste. Though they may accumulate in a manner essentially morbid, by displacing other structures, and though their presence in the body is not always limited to a healthy amount, still in proper quantity they are nutrient, and not merely indirectly aliment, as for instance serving, as they probably do, the purpose of respiatory food. Their disappearance is not by elimination, but by being consumed in the destructive changes of the body, as when it is deprived of sufficient nourishment from disease or other circumstances.

These considerations create a presumption against the idea that alcohol is directly food. It is true, it may in some way produce nervous force, and as the motive power of the organism, i. e. force is the ultimate aim and action of food, therefore it may in this sense be called food or generator of force.

Dr. Inman of Liverpool goes further than most others in his statement of the action of alcohol. He not only asserts that it is directly food, being incorporated with the tissues, and but a small portion appearing again by elimination, but he thinks it highly probable that the body manufactures alcohol for its own use. Saccharine material is found in the blood of mammals before it enters the lungs, and there disappears. This fact suggests to him the strong probability that by a process of fermentation it is converted into alcohol and carbonic acid in the body, as it is by fermentation out of it. In this way he would account for some of the carbonic acid which is exhaled by the lungs, the alcohol which is found at the same time being appropriated to the nourishment of the tissues. In other words, "Nature has provided in the salivary glands, the liver, and the lungs of every mammal, an apparatus for converting all food, especially farinaceous, into alcohol; and we have no evidence that such conversion does not take place." This, if true, makes alcohol far less alien to the human body than our teetotal friends would be willing to admit, and truly deals a heavy blow to their favorite theory of its poisonous action in all quantities. But fortunately they are saved from most unjust imputations, by the fact that in this case it does not obey the law of elimination by the lungs, as we have stated it. Were it so, we should be at a loss to distinguish alcohol of the body's own making, exhaled in the breath, from that taken into it through the stomach.

But alcohol, though it cannot properly be called food, in the sense of nourishing, i. e. in the building up or constructive sense, yet it replaces a certain amount of it by retarding destructive changes. Disintegration of tissue is much less rapid when alcohol is taken, and therefore less food is required. It is therefore conservative; it prevents waste if it does not contribute to repair. It is the equivalent of food, but not food. If alcohol is not taken, its place must be supplied by something else, starch in some form, either bread or pudding. Liebig states that it was found in temperance families, that the stopping of beer increased the consumption of bread. Also that the Peace Congress which met at Frankfort being made up largely of teetotalers, created daily at table, a deficiency in puddings and other starchy dishes, to the surprise of the landlord who had always accurately judged the needed quantity of such dishes, for a given number. They made up in pudding what they did not get in wine. Dr. Inman states that on inquiring into the habits of total abstainers and those who used alcohol in some of its forms, he found that the former ate much more than the latter, and he was led to the conclusion that either the former ate too much and the latter too little, or the drink of the one was equivalent to the food of the other.

In order to show that this is really the prominent action of alcohol, we will a little further on give the results of experimental researches upon the subject. Thus far we have considered the question of its passage through, and its final disappearance from the body. We have seen that it passes through unchanged, and whatever action it exerts it must act as alcohol, i. e. by presence. We have seen in what sense it is entitled to be ranked as food. As to the question of its being a poison we must admit its poisonous effects in excess, but not when used in moderation. It is confessedly not without a risk of some deleterious influence, even in quantities which might be called moderate, which effects will be spoken of by and bye. But the main argument that it is a poison in all quantities, because it is admitted to be poisonous in large, is entirely fallacious. In moderation, it will not only not be difficult to show that it is not a poison, but we may attribute to it positive advantages.

In further consideration of the actions of alcohol we may arrange them as follows:—First, we may consider its stimulant and the succeeding and opposite, sedative action. Secondly, its opposite effects upon nutrition, namely, to increase and diminish it. Thirdly, its opposite effects on metamorphosis of tissue, viz: to promote and retard it. Even in moderate use it exhibits that double action which is so marked as the result of small and large quantities, though this is more evident in that doubtful amount which is not quite moderate, but yet is rather short of excessive. The stimulant action of alcohol is familiar to all, too familiar to need much description. All know how it increases the activity of the mind and the energy of the muscular movements. The glow upon the countenance, the brightened eye, the warmth of surface and the increase of pulse are familiar to all. All know how it causes the timid to despise danger, the taciturn to become loquacious, and the despondent cheerful. Nor does it determine itself in opposites, but makes the witty more witty, the eloquent more eloquent, and the amiable more amiable. At the same time that it multiplies virtues, it may increase defects, so that he who is disposed to quarrel shall be more quarrelsome, and he who is noisy more boisterous. It may be said in its praise that it more often disposes to cheerfulness than despondency, and therefore is productive of a happy frame of mind. This stimulation in moderation is got without much exhaustion. There is always some sedative influence which succeeds, the more the greater the stimulation. But the sedative action of a large quantity may be so great without previous excitement that it produces depression, insensibility, coma and death. Instances of speedy death from large doses of alcohol are by no means rare. If the stimulation is excessive and frequent, the nervous system becomes disordered, the mind wanders in delirium, and the muscles are affected with spasm. Elimination sets all right again if time is given, and alcohol withheld.

The effects of alcohol on nutrition are of a double nature. It does not directly nourish, but by its invigorating influence upon digestion it produces the assimilation of a greater amount of food, and hence improves nutrition. In this way it may be said to nourish the body—it enables it to appropriate an increased amount of assimilable material to repair its waste. But its long continued use if in excess injures the stomach, thereby destroying the digestion; then the appetite is lost and the body wastes. Life may be maintained for a long time when little food, and much alcohol is taken, but it is a state of bodily decrepitude and mental imbecility. The tendency to the accumulation of fat in such cases, even when the limbs are attenuated is relied upon as a sort of evidence that alcohol is respiratory food, viz: it offers itself to be burned instead of the fat; but it is better explained by its effects to retard waste of tissue.

The third method of action of alcohol is in connection with the metamorphosis of tissue. Its double action is not as apparent here. As it stimulates secretions, so it may for a time stimulate excretion, i. e. promote waste. There are those who maintain that this is its action in disease, and that by the increased activity of waste under the stimulation of alcohol, the body supplies itself with food during sickness, i. e. feeds upon itself. But experimental facts do not confirm such a theory. They show the opposite. They show that small quantities of alcohol produce a decrease in the excretions which contain the waste of important tissues. There is a diminution of urine, of feces, and of carbonic acid exhaled by the lungs. The urine is diminished in quantity $\frac{4}{10}$ of the average amount. Its solid constituents moreover undergo a decrease relatively to the amount of urine. Urea is decreased $\frac{4}{10}$ in amount, uric acid $\frac{1}{7}$, the earthy phosphates $\frac{1}{6}$. Urea and uric acid represent the waste of the nitrogenous tissues, the phosphates of the nervous and bony structures. Alcohol is defensive to these important tissues, and hence a source of strength. If by a moderate amount of alcohol the muscles and the nervous system are spared waste, it is easy to perceive how with less food the body maintains itself. It becomes clear how the laborer may with a small amount of alcohol do more work and suffer less wear than without it; how the brain can be harder worked and suffer less exhaustion than without it. In fact it enables man to use his own body for a certain amount of extra work, bodily or mental, by husbanding its substance, and in the end be none the worse for the wear. We do not mean to say that in many cases food would not do as well, i. e. that it would not do as well instead of decreasing the waste, to increase the repair. All that we claim now is, that alcohol will limit waste, therefore prevent exhaustion, therefore render man stronger and fitter for social duties and labors. Other things may do the same.

The effect of alcohol is to limit the amount of fecal matters and hence produce constipation, an effect of its habitual use often observed. This is due partly to its retarding the escape of water, and thus rendering the fecal matters dry and hard; and partly to its limiting the amount of substance arising from the destructive waste, to be got rid of that way, as the intestines are an important outlet for the escape of effete tissue.

More remarkable is the decrease of the amount of carbonic acid exhaled by the lungs. The diminution amounts to one-fifth the average amount. This gives the final blow to the theory of the combustion of alcohol by the lungs. It is not in any sense a supporter of combustion ; rather, by the diminution of carbonic acid, it retards in the body the destruction of carbon by oxygen.

Thus alcohol for a time at least exerts a conservative action upon the body, not by chemical combination, not by offering itself to the destructive action of oxygen, i. e. by combustion, not as a pabulum to the tissues, but by retarding their waste. In what way except by presence as alcohol, is all that can be stated. The physical agencies by which it effects it are in obscurity.

But though muscular activity wears out the body and alcohol is protective against its wasting agency, it is not the most important nor the most interesting of its operations. Brain work not only wears out the brain, but wastes the material fabric with which it is connected. The destructive energy of the mind "frets to dust its tenement of clay." All mental operations, all emotions, care and sorrow, anxiety, as well as the nobler aspirations of the scholar or the patriot, all act with wondrous energy to destroy the bodily frame. Alcohol then protects the body against the mind, and its beneficial operation in this is most apparent and most needed in those spheres of life where mental effort and anxiety are greatest, as in cities, where many causes tend to exhaust the nervous system, and hence wear out the body.

But there is another view of the question which must not be put out of sight. As far as alcohol is protective and nothing more, it is a fact in its favor, but it must not be forgotten that this advantage is not got without the chance of harm—therefore at the risk of too great a cost. We must admit that its use even in moderation is attended with the chance that its retarding influence on tissue charge may pass the bounds within which it is salutary—and thus effete and worn out materials may be retained within the body. From this, results degeneration of tissue and disease. When the use of a substance, which is attended with positive advantages, carries with it a liability of such a bad result, there is a great difficulty in so using it, as to get only the good and run clear of the evil. This is not an imaginary danger. When any alcoholic liquor, as, for instance, brandy, is taken habitually, even in moderation, the blood is soon found to deviate in its composition from that of healthy blood. Its solid constituents are diminished. It is deficient in fibrine, has a more carbonized clot, like venous blood, and contains in excess colorless corpurcles, i. e. bodies which are supposed to have lost their power of absorbing oxygen and giving out carbonic acid; in other words, worn out blood globules. Hence the blood has less vital properties and is deficient in tissue-forming material. Abounding in worn out material, it loses it capability for healthy repair. The tissue of the organs thus being not healthily constituted, disturbance of their functions follows and the various diseased conditions which are the well known results of excessive use begin.

This is but the beginning of that pathological change of the blood, which, in its more advanced state, is thus described by Rokitansky: "In the dyscrasial condition induced by the abuse of alcoholic drinks, the blood appears dark colored, grumous, defibrinated, viscid, unctuous to the touch, often intermingled with fat in large quantity, as fat drops. In rare instances the disease occasions a chyle-like opacity of the plasma—milky blood." But this, though a true picture, is not so pertinent to our subject, viz: rather to consider the advantages and evils of alcohol in moderation, for in excess we do not deny the evils, both moral and physical which spring from its use.—We are to satisfy ourselves, if we can, that there are advantages attending its moderate use which justify its recommendation. Into the question of the morbid organic changes it causes in excess, it is not worth while to enter.

I may in conclusion speak of the moderate habitual use of alcohol in health, and its advantages in disease. It may be accepted as true that alcoholic drinks as articles of regular daily consumption are, to many persons, of great benefit; and to others, necessary. They are more needed by those whose occupations require study, or involve anxiety or excitement.

The wear and tear of brain is not so easily repaired as that of muscles. In proportion as the labor is intellectual is the necessity for an alcoholic stimulus felt, as well as varied and generous food. As Dr. Hooper expresses it, "Men whose labor resembles that of horses may and do live like horses —upon corn and water; but those who are calculating, thinking, reasoning twelve hours out of the twenty-four require a more refined sort of food and drink." It is capable of proof, that the variety and, as it is called, luxury of modern tables, used in moderation, has conduced to longevity as well as vigorous health, and in this way prevented disease. But gluttony, as well as drunkenness, is ruinous to body and mind.

As far as the moderate use of alcohol is a medical question it should not be mixed up with affairs pertaining to temperance. Its habitual use is to be recommended with a full sense of responsibility, and only to help towards that most desirable thing, the procuring for the use of the mind the soundest possible body. Apart from such a consideration, or as an indulgence, its habitual use is likely to be michievous. But in civilized life it cannot be denied that there are many persons whose functions, bodily and mental, are better performed and whose usefulness is increased by a moderate daily allowance of some alcoholic drink. The question is correctly stated by Dr. Chambers. Its benefits are to be judged of, by its effects during those hours between meals in which digestion is going on. If the body during this period is more active, more efficient for the mind's purposes when alcohol is taken than when it is not, we may feel sure that the individual, whether so-called healthy or invalid, is benefited by it.

We are not prepared to admit that in the perfectly healthy body it is ordinarily beneficial or necessary. At the same time we must admit that it is extremely difficult to define what health is, i. e. perfect regularity in the performance of the functions of the body. The necessity which our digestion imposes of careful preparation of our food, by cooking, is an indication of our departure from the perfection of bodily vigor.— Hence the need of being saved from the consequences of our deteriorated condition, debility or disease, by some artificial aid to enable the body to get the most out of the substances which restore it, to strengthen it against wear and tear. The question is simply what will give the greatest ability to man to convert food into blood and flesh; or, failing of that, to retard its wasting by its own destructive agencies. Alcoholic stimulants perform this useful part. But it certainly is desirable that for this purpose their use.

should be temporary and not habitual, if the end can be so answered. And upon the whole, we believe they are more proper to extricate us from danger than to contribute to our support. For it is not to be denied that we gain our advantage from moderate use, at the peril of running into all the terri le sequences of excess. Moreover even that moderate use which is hard to define, by its effects upon the blood, endangers the object it seeks. So much for the use of alcohol, either temporary or habitual, in what is ordinarily called health. It cannot probably be claimed that alcohol increases in the healthy body the capability of enduring fatigue or cold, or of resisting disease, at least for any length of time. Temporarily it may do so, but habitual use rather diminishes than increases its endurance of prolonged and continuous exertion, whether in soldiers, explorers in warm regions, or arctic navigators.

The use of alcoholic liquors in disease is a subject of much greater importance to medical men. We are accustomed to speak of them as stimulants and to associate with their operation an idea of exaltation of vitality, or as it is often expressed vaguely, nervous power, rather than of a defensive or conservative influence under the wasting effects of disease. From what has been said of their influence to retard metamorphosis of tissue, we are prepared to find them efficacious in all cases in which waste is more active than repair. This is the case in many diseases, both acute and chronic, but there is this difference, that in acute diseases waste is usually only normal, while in many chronic cases, especially those in which active suppuration is going on, destruction is excessive. If then from the circumstances of the case the supply of nutriment is limited, as in acute disease, repair cannot make up even normal waste. Alcohol by retarding waste tends to equalize repair and destruction.

This seems to be its modus operandi in acute diseases, and certainly though the practice may have been carried to extremes, the results have been in the highest degree beneficial. No one doubts the efficacy of the practice in typhus or typhoid fevers, but many are sceptical of its benefits in pneumonia, pleurisy, and other acute diseases, and in surgical injuries, which have been heretofore subjected to the depleting method. Even in these last experience will yet show the superiority of the treatment by stimulants, which is, if our reasoning is correct, conservative. No doubt quantities too large are often given, so that the stupor of disease is not easily distinguished from the sedative effects of alcohol. Such liabilities should be avoided by giving small quantities, which by rapid elimination are not likely to produce such an effect. To these cases may be added those in which from feebleness of the digestive organs nutriment enough cannot be taken to supply the ordinary waste. In such cases alcohol is of benefit, and perhaps by a double operation, exalting as well as conserving. In chronic cases in which destruction is abnormally active, and we may reckon phthisis one of the most important of them, the only rational course is to render repair as far as possible compensatory for the excessive waste. Normal repair is unequal to the work, as is often most painfully evident to us; and even such aid as we can procure in alcohol too often fails. But it is one of the agents for the procuring of such a desirable aim which we should not hesitate to call to our aid.

